# Efficacy and Safety of Immunotherapies in Refractory Myasthenia Gravis: A Systematic Review and Meta-Analysis

**DOI:** 10.3389/fneur.2021.725700

**Published:** 2021-12-01

**Authors:** Xuelin Feng, Zubiao Song, Mengli Wu, Yanmei Liu, Sushan Luo, Chongbo Zhao, Weixi Zhang

**Affiliations:** ^1^Department of Neurology, East Hospital, Tongji University School of Medicine, Shanghai, China; ^2^Department of Neurology, National Key Clinical Department and Key Discipline of Neurology, The First Affiliated Hospital, Sun Yat-sen University, Guangzhou, China; ^3^Department of Neurology, Huashan Hospital, Fudan University, Shanghai, China

**Keywords:** refractory myasthenia gravis, immunotherapies, tacrolimus, eculizumab (monoclonal antibody to C5), rituximab-ofatumumab-ibrutinib-idelalisib

## Abstract

**Introduction:** Approximately 10–20% of patients WITH myasthenia gravis (MG) are refractory to conventional immunotherapies. The purpose of this study was to conduct a systematic review and meta-analysis to explore the optimal therapies for refractory MG.

**Method:** Correlative studies were performed through a search in PubMed, Cochrane Library, and Embase databases. The primary outcome was defined by changes in the quantitative myasthenia gravis score (QMG). Secondary outcomes were defined by the Myasthenia Gravis Activities of Daily Living Scale (MG-ADL), Myasthenia Gravis Foundation of America (MGFA) post intervention status, adverse events, and disease exacerbation after treatment.

**Result:** A total of 16 studies were included with 403 patients with refractory MG on therapies with rituximab, eculizumab, tacrolimus, and cladribine. Therapeutic efficacy of rituximab and eculizumab was identified with an estimated reduction in QMG score (4.158 vs. 6.928) and MG-ADL (4.400 vs. 4.344), respectively. No significant changes were revealed in efficacy or exacerbation density between the two independent therapeutic cohorts. The estimated adverse event density of eculizumab was more significant than that in the rituximab group (1.195 vs. 0.134 per patient-year), while the estimated serious event density was similar.

**Conclusion:** The efficacy and safety of rituximab and eculizumab have been approved in patients with refractory MG. Rituximab had a superior safety profile than eculizumab with a lower incidence of adverse events.

**Systematic Review Registration:** https://www.crd.york.ac.uk/prospero/display_record.php?ID=CRD42021236818, identifier CRD42021236818.

## Introduction

Myasthenia gravis (MG) is an autoimmune disorder due to a transmission defect in the neuromuscular junction. These patients clinically manifest with fluctuating muscle weakness in an ocular, limb, and axial muscles. The majority of patients with MG have excellent responses to acetylcholinesterase inhibitors, rescue therapies (such as intravenous immunoglobulin and plasma exchange), immunosuppressants including glucocorticoid, azathioprine, mycophenolate mofetil, and thymectomy (1). However, still, a substantial proportion of patients have poor responses to conventional immunotherapies, termed refractory MG. Patients with refractory MG are usually managed with long-term high-dose glucocorticoid or immunosuppressive agents, which are associated with severe adverse events (2). In the recent decades, emerging clinical trials have been conducted using cyclophosphamide, rituximab, and eculizumab (3–5) to treat patients with refractory MG. Currently, the guidelines for therapeutic options in refractory MG have not been adequately established in clinical practice. In this study, we performed a meta-analysis to evaluate and compare the efficacy and safety of immunotherapies for patients with refractory MG.

## Methods

### Protocol Registration

The meta-analysis protocol has been registered in PROSPERO (International Prospective Register of Systematic Reviews) under ID: CRD42021236818.

### Search Strategy

One author (FX) performed an article search in PubMed, Cochrane Library, and Embase. Medical subject headings (MeSH) and free-text words were used for PubMed and Cochrane: Myasthenia Gravis/therapy (MeSH) and refractory. In Embase, Emtree, and free-text words were used: MG (Emtree), therapy (Emtree), and refractory. The primary search was completed on December 16, 2020 and the final search was completed on March 15, 2021. Only English language papers were included.

### Study Selection

**Figure 1:**
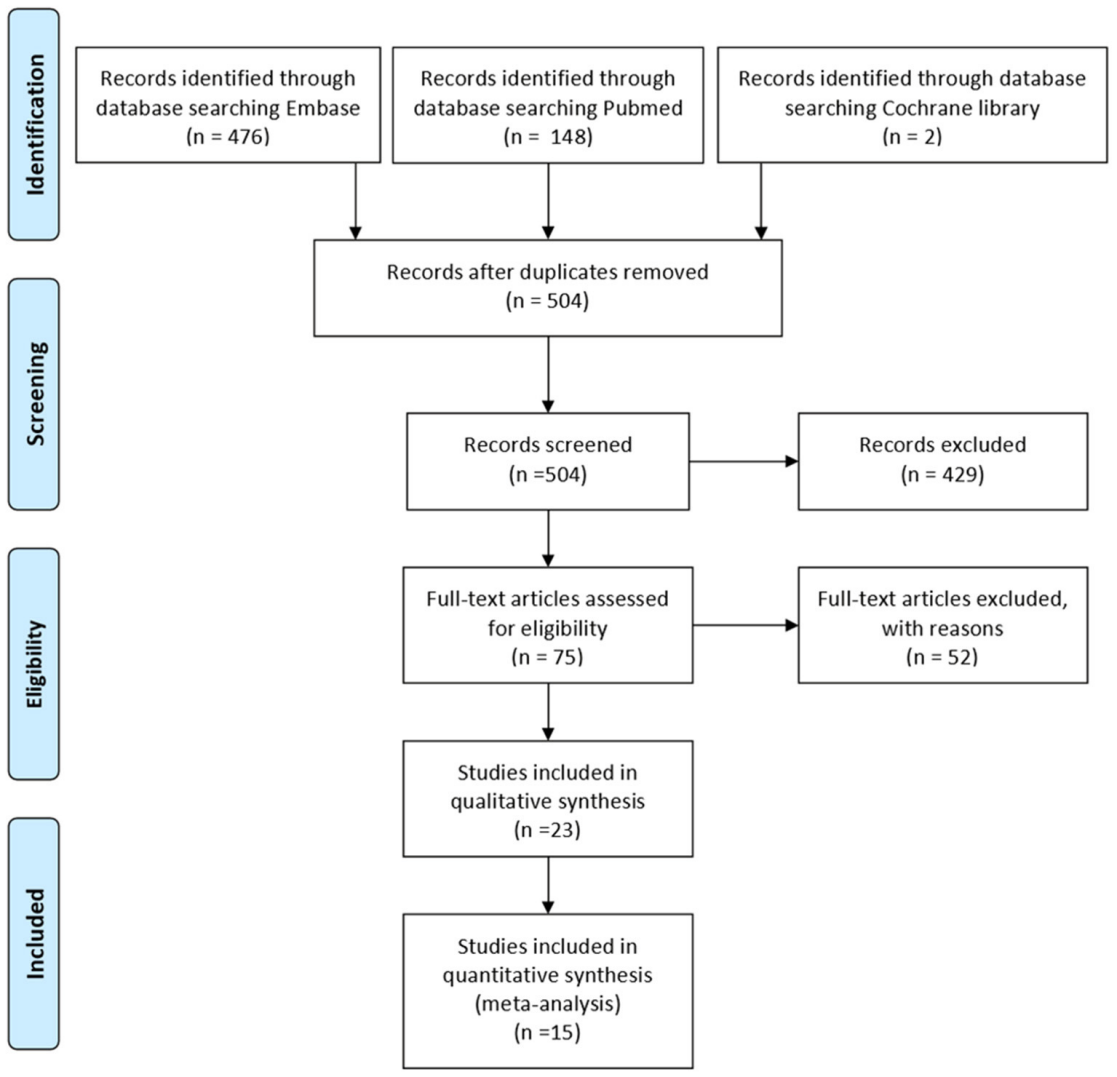
The preferred reporting items for systematic reviews and meta-analyses (PRISMA) flow chart of studies selection.

Randomized controlled trials and observational studies with at least 10 patients with MG with each intervention were included. Refractory MG was defined according to criteria of Mantegazza (6): 1. Patients have an insufficient response to maximal safe doses of steroids and at least one immunosuppressive drug at an adequate dose and duration, 2. Inability to reduce immunosuppressive therapy without clinical relapse or a need for ongoing rescue therapy such as intravenous immunoglobulin or plasma exchange, 3. Severe or intolerable adverse effects from immunosuppressive therapy, 4. Comorbid conditions that restrict the use of conventional therapies, 5. Frequent myasthenic crises even while on therapy. Exclusion criteria included: 1. Studies that did not have a clear standard of refractory MG or failed to meet criteria of Mantegazza, 2. Studies that did not evaluate the efficacy of specific therapy with a quantitative outcome, 3. Studies that were restricted to ocular MG or juvenile MG. Included studies were first filtered based on the title and abstract. Further assessment for eligibility was based on the full text of the studies (PRISMA flow chart; Figure 1).

### Quality Evaluation

Newcastle-Ottawa Scale (NOS) was applied for quality evaluation for observational studies (7). According to the Cochrane Handbook, randomized clinical trials and non-randomized controlled studies were assessed (8). Two authors (FX and SZ) independently evaluated the quality of the included studies, and an open discussion would be held to resolve disagreements.

### Data Extraction

Two authors (FX and WM) independently extracted data. The discussion aimed to resolve any disagreements until a consensus was reached, or by consulting a third author (ZW). The following data were extracted: author, year of publication, country, original inclusion criteria, the total number of patients included in the study, intervention, related quantitative outcomes, and adverse events. Serious adverse events include adverse events that are life-threatening or result in death, hospitalization, or persistent or significant disability or incapacity, are congenital anomalies or birth defects, or are essential medical events (9). Worsening of MG and MG crisis were not considered as serious adverse events.

### Outcome Definition

The primary outcome was the changes in the quantitative myasthenia gravis score (QMG) (10). Secondary outcomes include Myasthenia Gravis Composite (MGC) scale (11), manual muscle test (MMT) (12), Myasthenia Gravis Activities of Daily Living (MG-ADL) scale (13), Myasthenia Gravis Foundation of America (MGFA) post intervention status (PIS) (14), adverse events and exacerbation of MG (defined as MG symptoms with increased frequency and/or intensity), and myasthenic crisis (defined as exacerbated MG symptoms that required intubation or rescue therapy such as plasma exchange and intravenous immunoglobulin) during treatment.

### Statistical Analysis

Statistical analysis was based on STATA (version 14 for Windows). If some of the included studies failed to provide the SD of the change of reported outcomes, we would calculate it based on the methods described by The Cochrane Collaboration in the Cochrane Handbook for Systematic Reviews of Interventions (section 6.5.2.8, the correlation coefficient was calculated from similar studies) (8). *Q*-test and *I*^2^-statistics were used for heterogeneity analysis. Fixed effect model or random effect model will be used based on heterogeneity (random effect model will be used if *I*^2^ > 50%). Publication bias was explored with Egger's test.

## Results

### Search and Selection Results

Our search in Embase, PubMed, and Cochrane library provided 626 studies. After deduplication, 504 studies were screened for the title and abstract. Full-text assessments were then performed for 75 studies, and we subsequently included 23 studies. Among them, seven studies did not report main outcomes, additional outcomes, or other quantitative outcomes of treatment effect. Therefore, 16 studies finally entered the quantitative synthesis (5, 15–26) (Figure 1). Of these included studies, 13 were observational studies and two were randomized controlled trials. The remaining was an open-label extension of other included studies (17). Quality evaluation of the included studies was in Supplementary Table 1. A summary of all the full-text reviewed studies was in Supplementary Table 2.

### Overall Features of Included Studies

In the included studies, eight studies were on rituximab and five studies were on eculizumab, while two studies were about tacrolimus and cladribine, respectively (Table 1). In these studies, 403 patients (254 females and 149 males) were included (Table 2). The mean age of overall patients at recruitment was 48.4 ± 19.06 years. The mean age of the patients in studies categorized by different therapies was 49.3 ± 15.97 (rituximab), 48.6 ± 16.59 (eculizumab), 43.8 ± 13.20 (tacrolimus), and 60.8 ± 18.59 (cladribine), respectively. The mean age of the cladribine group was significantly higher than rituximab (*p* = 0.0069, Bonferroni corrected) and tacrolimus group (*p* = 0.0013, Bonferroni corrected). There were 339 cases with the antiacetylcholine receptor (AChR) antibody positive and 43 cases with muscle-specific tyrosine kinase (MuSK) antibody positive. All the cases with positive anti-MuSK antibodies were recruited in studies about rituximab. The remaining 21 patients were seronegative for MG-related antibodies.

**Table 1:** Summary of all the selected studies.

**No**.	**Author**	**Year**	**Therapy**	***N***	**AChR-ab +**	**Musk-ab +**	**Seronegative**	**Outcomes**
1	H. Wu	2020	Tacrolimus	24	18	0	6	QMG, MG-ADL
2	M. Oyama	2020	Eculizumab	11	11	0	0	QMG, MGFA-PIS, MG-ADL
3	T. Levine	2019	Eculizumab	13	13	0	0	MGC
4	J. Howard^*^	2017	Eculizumab	62	62	0	0	QMG, MG-ADL
5	R. Mantegazza^*^	2021	Eculizumab	117	117	0	0	MGFA-PIS
6	J. Howard	2013	Eculizumab	14	14	0	0	QMG, MG-ADL
7	R. Govindarajan	2020	Eculizumab	15	15	0	0	MG-ADL
8	D. Anderson	2016	Rituximab	14	5	6	3	MMT
9	k. Choi	2019	Rituximab	17	9	6	2	MGFA-PIS
10	S. Jing	2019	Rituximab	15	13	1	1	QMG, MGFA-PIS, MG-ADL
11	R. Topakian	2019	Rituximab	56	39	14	3	MGFA-PIS
12	O. Landon-Cardinal	2018	Rituximab	11	11	0	0	QMG
13	K. Robeson	2017	Rituximab	16	16	0	0	MGFA-PIS
14	T. Litchman	2020	Rituximab	33	17	16	0	MGFA-PIS
15	S. Brauner	2020	Rituximab	34	28	0	6	QMG
16	K. Rejdak	2020	Cladribine	13	13	0	0	MGC

* *Study 5 was the open-label extension of Study 4. AChR, acetylcholinergic receptor; Musk, muscle-specific tyrosine kinase; MG-ADL, Myasthenia Gravis-Activities of Daily Living; MGC, Myasthenia Gravis Composite; MGFA-PIS, Myasthenia Gravis Foundation of America post-intervention status; MMT, manual muscle test; QMG, Quantitative myasthenia gravis*.

**Table 2:** Clinical characteristics of patients treated with rituximab, eculizumab, tacrolimus, and cladribine.

**Therapy**	**Rituximab**	**Eculizumab**	**Tacrolimus**	**Cladribine**
No. of studies	8	5	1	1
Patients	196	170	24	13
Gender, female,%	63%	65%	67%	38%
Age at treatment	49.3 ± 15.97	48.6 ± 16.59	43.8 ± 13.20	60.8 ± 18.59
AChR-ab+	138	170	18	13
Musk-ab+	43	0	0	0
Seronegaitve	15	0	6	0

*Ab, antibody; AChR, acetylcholine receptor; Musk, muscle-specific tyrosine kinase*.

### Efficacy in Ameliorating Disease Severity

**Figure 2:**
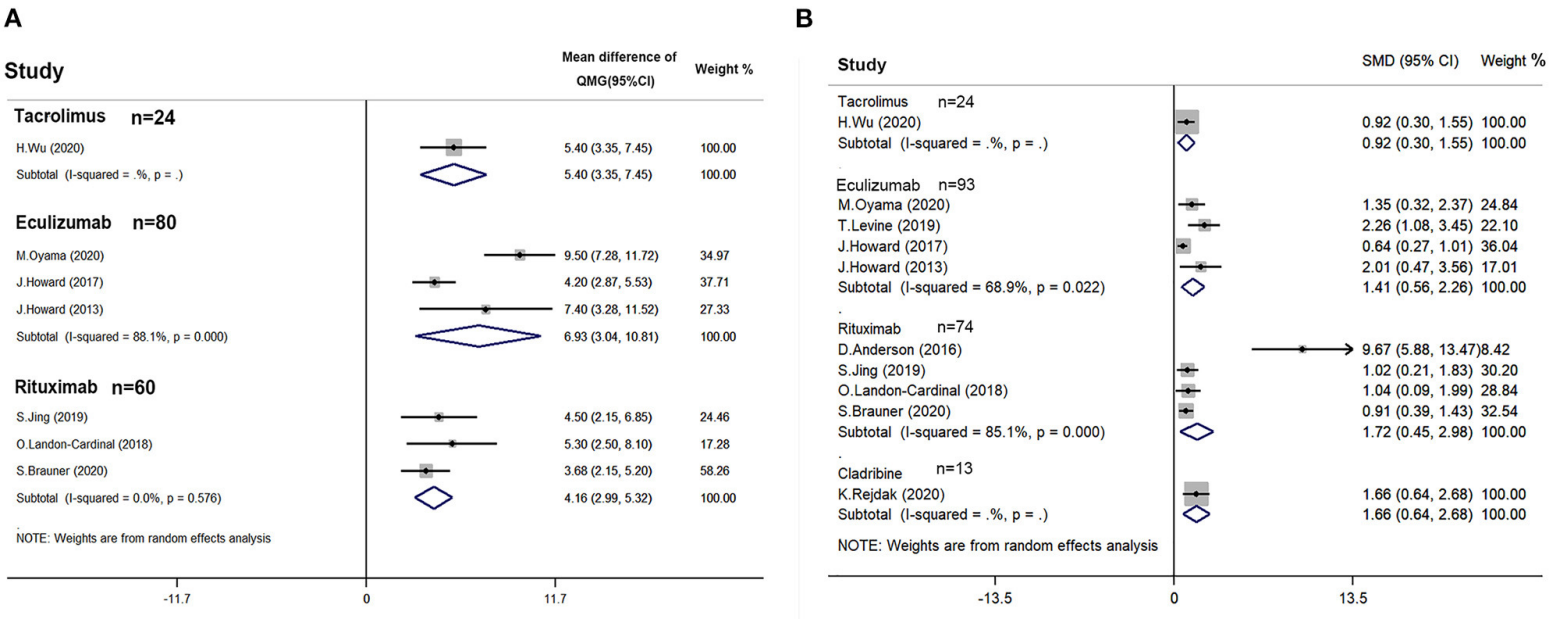
Efficacy of immunotherapies in refractory myasthenia gravis (MG) based on quantitative myasthenia gravis score (QMG). **(A)** Quantitative synthesis of QMG reduction of rituximab, eculizumab, and tacrolimus. **(B)** Quantitative synthesis of severity index reduction based on QMG of rituximab, eculizumab, tacrolimus, and cladribine [Myasthenia Gravis Composite scale was used for studies of Levine (27) and Rejdak et al. (20). Manual muscle test was used for the study of Anderson et al. (28)]. A random-effect model was used for quantitative synthesis. Note that each therapy relieved the symptom of refractory myasthenia gravis (MG). However, no significant difference in therapeutic efficacy among these therapies. SMD stands for the standard mean difference.

In the included studies, eight studies provided the primary outcome data in the QMG score (Figure 2A). The study by Jing et al. failed to provide the SD of the change of QMG score. It was imputed with the average correlation coefficients of Landon-Cardinal et al., and Brauner et al. Random effect model was used for quantitative synthesis. The pooled mean difference of rituximab was 4.158 (95% CI: 2.994–5.323). The pooled mean difference of tacrolimus was 5.400 (95% CI: 3.350–7.450). The pooled mean difference of eculizumab was 6.928 (95% CI: 3.042–10.813). No small-study effect was found by Egger's test (*p* = 0.281).

Two of the included studies provided an MGC scale (20, 27), and one included a study that provided MMT other than QMG score (28). Combined with these studies, a standard mean difference in Glass's Δ was calculated in the random effect model (Figure 2B). The estimated standard mean difference of rituximab was 1.719 (95% CI: 0.453–2.985). The estimated standard mean difference of eculizumab was 1.409 (95% CI: 0.556–2.262). The estimated standard mean difference of tacrolimus and cladribine were 0.923 (95% CI: 0.297–1.548) and 1.66 (95% CI: 0.64–2.68), respectively. However, a small-study effect was found by Egger's test (*p* < 0.001).

Six of the included studies had reported MG-ADL results (Supplementary Figure 1). The study by Jing et al. failed to provide the SD of the change of MG-ADL score. It was imputed with the correlation coefficient as 0.5. Fixed effect model was used for quantitative synthesis. The effect size was calculated with standard error. The pooled mean difference of rituximab was 4.400 (95% CI: 2.610–6.190). The pooled mean difference of tacrolimus was 3.330 (95% CI: 1.839–4.821). The pooled mean difference of eculizumab was 4.344 (95% CI: 3.944–4.744). No small-study effect was found by Egger's test (*p* = 0.706).

**Figure 3:**
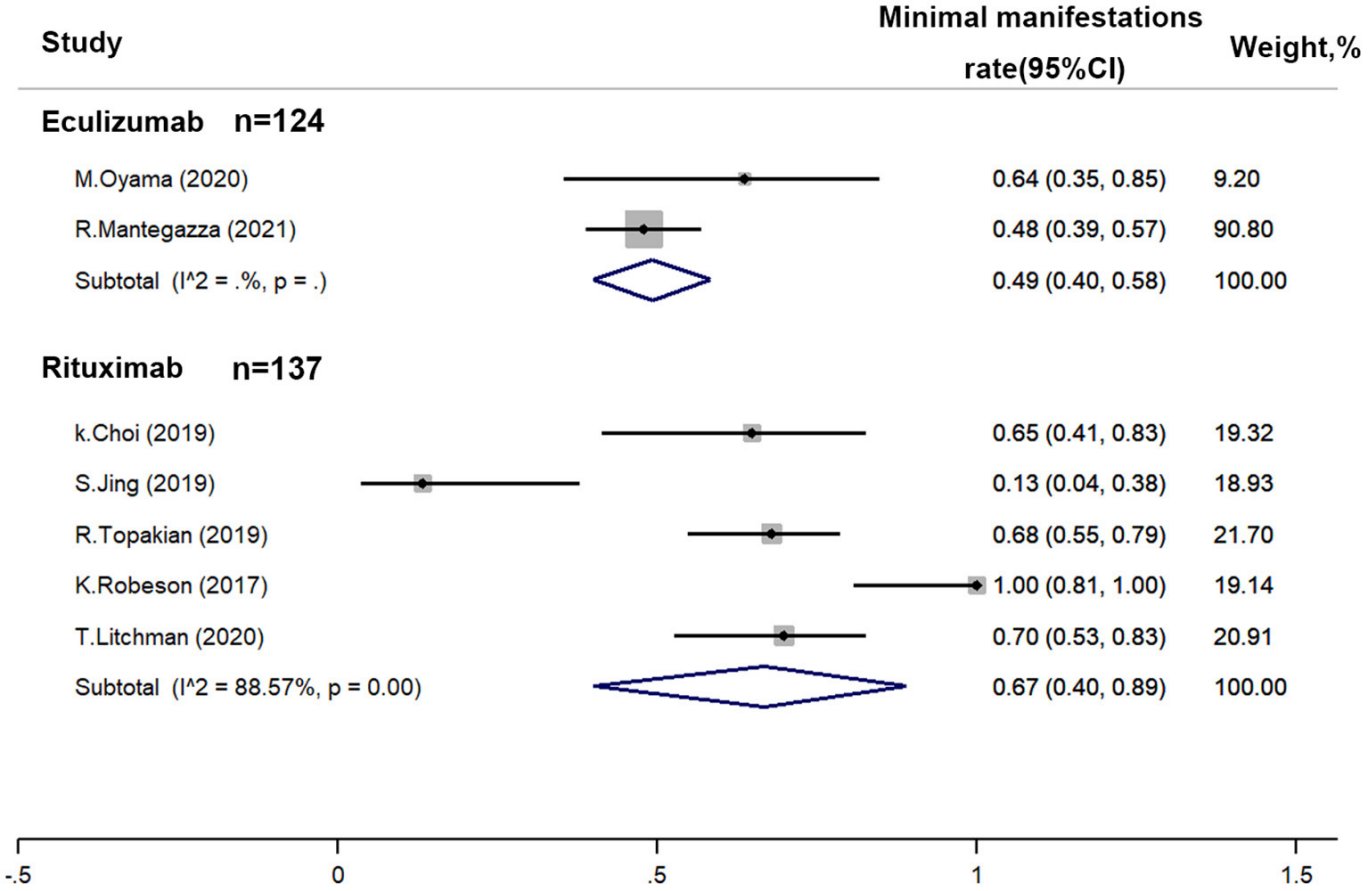
Efficacy of immunotherapies in refractory myasthenia gravis based on the achievement proportion of minimal manifestations (MM) (Myasthenia Gravis Foundation of America post intervention status). A random-effect model was used for quantitative synthesis. No significant difference between the MM rate of rituximab and eculizumab was found.

Five of the included studies had provided the percentage of patients with MGFA-PIS as minimal manifestations (MM) (Figure 3). A random-effect model was used for quantitative synthesis. The estimated MM rate of rituximab was 67% (95% CI: 0.40–0.89), while the estimated MM rate of eculizumab was 49% (95% CI: 0.40–0.58). No small-study effect was found by Egger's test (*p* = 0.570). In addition, after the removal of Musk antibody-positive patients, the estimated MM rate of rituximab became 61% (95% CI: 0.28–0.90).

### Efficacy in Preventing Worsening or Fluctuation

**Figure 4:**
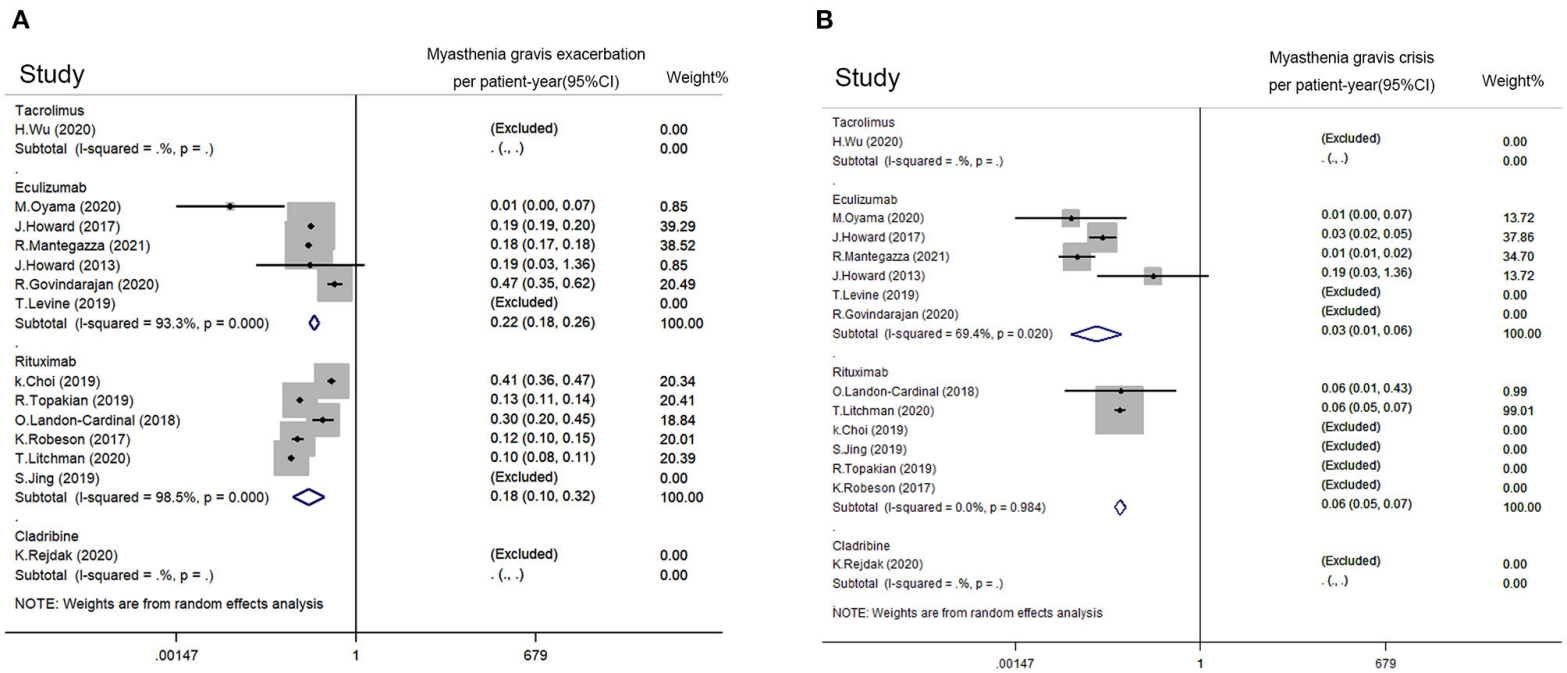
Impact from immunotherapies on refractory MG based on disease exacerbation. **(A)** MG exacerbation rate per patient-year of rituximab, eculizumab, tacrolimus, and cladribine. **(B)** MG crisis rate per patient-year of rituximab, eculizumab, tacrolimus, and cladribine. MG exacerbation was defined as MG symptoms with increased frequency and/or intensity. Myasthenic crisis was defined as exacerbated MG symptoms that required intubation or rescue therapy such as plasma exchange and intravenous immunoglobulin. A random-effect model was used for quantitative synthesis. No significant difference in frequency of MG exacerbation or crisis between the rituximab group and eculizumab group was revealed. The studies with Tacrolimus and cladribine were excluded since no disease exacerbation or crisis has been reported.

The incidence density of MG exacerbation and crisis was estimated with a random effect model. The estimated incidence density of MG exacerbation for rituximab was 0.178 per patient-year (95% CI: 0.099–0.319). The estimated incidence density of MG exacerbation for eculizumab was 0.218 per patient-year (95% CI: 0.182–0.262) (Figure 4A). No small-study effect was found by Egger's test (*p* = 0.822). The estimated incidence density of crisis for rituximab was 0.059 per patient-year (95% CI: 0.049–0.072). The estimated incidence density of crisis for eculizumab was 0.026 per patient-year (95% CI: 0.011–0.062) (Figure 4B). No small-study effect was found by Egger's test (*p* = 0.390).

### Common Adverse Events of Immunotherapies

**Figure 5:**
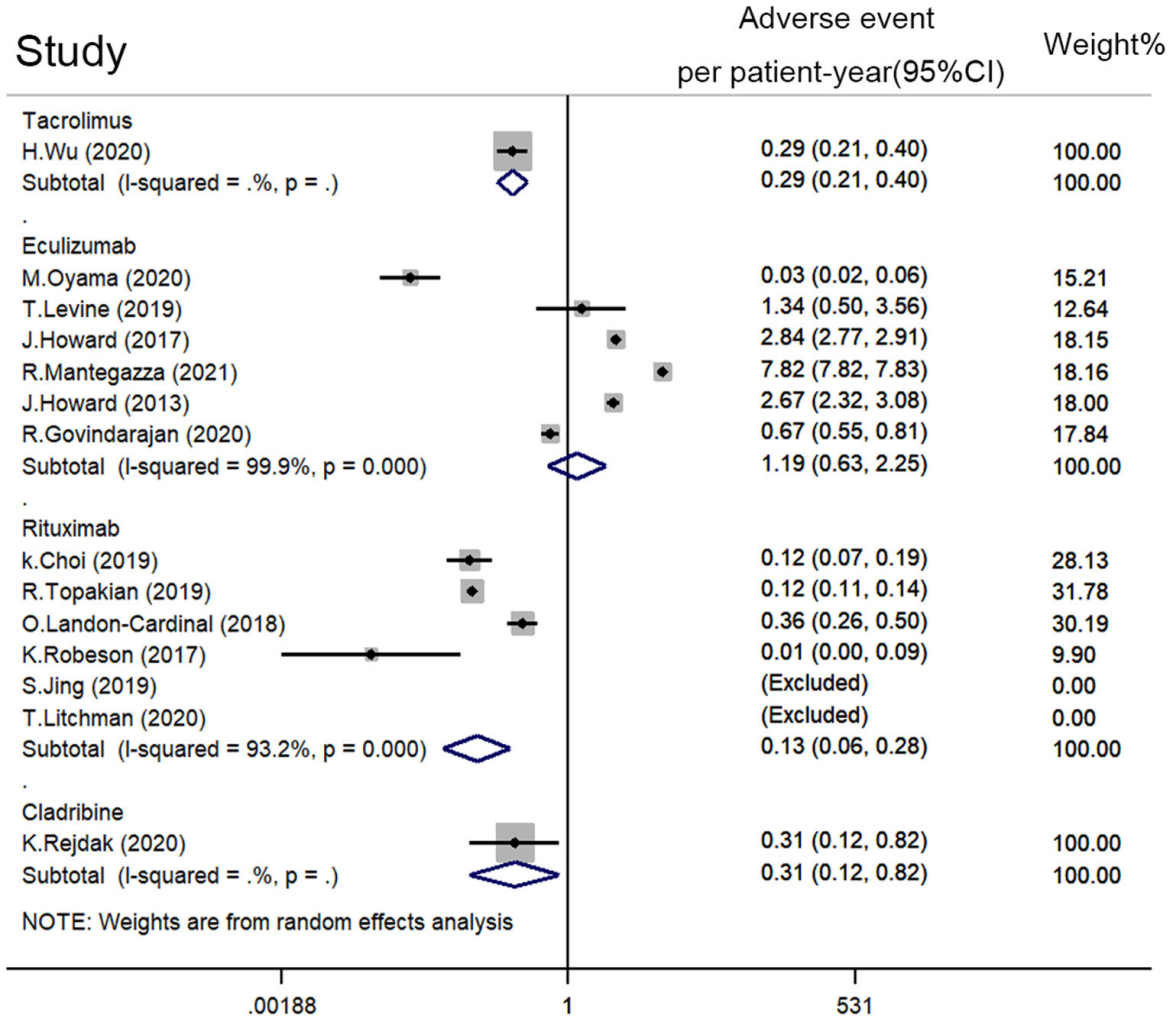
Adverse event density (event rate per patient-year) of rituximab, eculizumab, tacrolimus, and cladribine on refractory MG. A random-effect model was used for the quantitative synthesis. Note that eculizumab had more adverse events than rituximab and tacrolimus. Besides, no significant difference in adverse event density between rituximab, tacrolimus, and cladribine was revealed.

All included studies except for Brauner et al. reported adverse therapy events on patients with refractory MG. The incident density of adverse events and serious adverse events were calculated. A random-effect model was used for quantitative synthesis. The estimated adverse event density of rituximab was 0.134 per patient-year (95% CI: 0.064–0.281), while the estimated adverse event density of eculizumab was 1.195 per patient-year (95% CI: 0.635–2.248) (Figure 5). Tacrolimus and cladribine estimated adverse event densities at 0.292 per patient-year (95% CI: 0.210–0.404) and 0.308 per patient-year (95% CI: 0.115–0.820), respectively. A small-study effect was found by Egger's test (*p* = 0.009).

Common adverse events of eculizumab include headache (18%, 95% CI: 0.04–0.36), nausea (14%, 95% CI: 0.09–0.20), myalgia (11%, 95% CI: 0.06–0.17), nasopharyngitis (8%, 95% CI: 0.00–0.27), upper respiratory tract infection (7%, 95% CI: 0.00–0.21), and diarrhea (3%, 95% CI: 0.00–0.17). Common adverse events of rituximab include upper respiratory tract infection (1%, 95% CI: 0.00–0.06), infusion reaction (2%, 95% CI: 0.00–0.05), herpes zoster infection (1%, 95% CI: 0.00–0.04), and enteritis (1%, 95% CI: 0.00–0.04) (Supplementary Figure 2A).

The estimated serious adverse event density of rituximab was 0.082 per patient-year (95% CI: 0.035–0.190), while the estimated adverse event rate of eculizumab was 0.281 per patient-year (95% CI: 0.146–0.540) (Supplementary Figure 2B). No small-study effect was found by Egger's test (*p* = 0.058).

### Comparison of Low-Dose and High-Dose Rituximab

**Figure 6:**
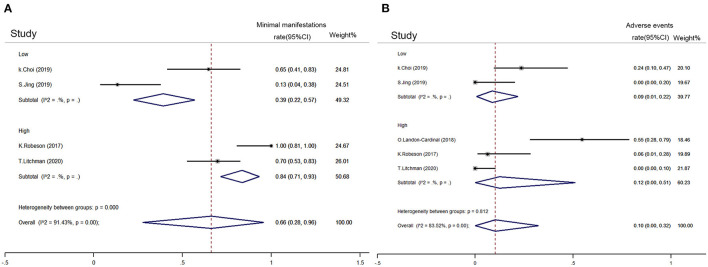
Comparison between refractory MG subgroups with low-dose and high-dose rituximab. **(A)** Achievement proportion of MM (MG Foundation of America post intervention status) between low-dose and high-dose rituximab on refractory MG. **(B)** Adverse event density (event rate per patient-year) between low-dose and high-dose rituximab on refractory MG. High-dose rituximab was defined as a dose higher than 375 mg/m^2^ twice in a month and vice versa. A random-effect model was used for quantitative synthesis. The high-dose rituximab had better performance in achieving the MM rate than low-doses with similar adverse event density.

The rituximab regimens included in the study were different. The low-dose rituximab was defined as a dose lower than 375 mg/m^2^ twice a month. Studies of low-dose rituximab included Choi et al. (29), Jing et al. (24), and Brauner et al. (21). Other regimens were considered as high doses. Studies of high-dose rituximab included Landon-Cardinal et al. (25), Robeson et al. (23), and Litchman et al. (22). The estimated MM rate of low-dose rituximab was smaller than that of high-dose rituximab (39%, 95% CI: 22–57 vs. 84%, 95% CI: 71–93, Figure 6A). The pooled adverse events rate of low-dose rituximab was 9% (95% CI: 1–22), and that of high-dose rituximab was 12% (95% CI: 0–32) (Figure 6B).

## Discussion

A subset of MG is refractory to standard immunosuppressive therapy, whereby appropriate treatment is still uncertain. A total of 13 observational studies and three controlled studies included in this study identified rituximab, eculizumab, tacrolimus, and cladribine as possible optimal treatments.

Eculizumab is a-C5 monoclonal antibody, which inhibits C5 cleavage (30) and prevents damage to the neuromuscular junction from complement cascade in MG patients with AChR antibody (31). Similarly, rituximab is a monoclonal antibody targeting CD20 antigen. It modulates B-cell activation and inhibits AChR antibody-dependent cytotoxicity in MG (32, 33). Tacrolimus and cladribine are non-steroidal immunosuppressants. Tacrolimus inhibits T cells and dampens antibody production (34). Cladribine has a selective effect on B and T lymphocytes, sustaining reduction of peripheral lymphocytes (35).

The efficacy of rituximab (4) and eculizumab (36) in MG has been proven in previous meta-studies. However, their efficacy in refractory MG has not been investigated yet. This study included 170 patients treated with eculizumab and revealed its efficacy for refractory MG. Quantitative synthesis showed that eculizumab relieved the symptoms of the patients refractory MG (estimated reduction of QMG: 6.93, estimated reduction of MG-ADL 4.34, estimated MM rate:49%, estimated incidence density of MG exacerbation: 0.218 per patient-year). Similar efficacy of rituximab was revealed in a study including 196 patients (estimated reduction of QMG: 4.16, the estimated reduction of MG-ADL 4.40, estimated MM rate: 67%, estimated incidence density of MG exacerbation: 0.178 per patient-year). However, no significant difference between their efficacy was found. Similarly, no significant differences between the incident density of MG exacerbation and also the incident density of MG crisis were found.

The estimated adverse event density of eculizumab was more significant than that of rituximab (1.195 vs. 0.134 per patient-year). However, no significant difference in adverse severe event density was found between them. The most common adverse events of eculizumab were headache (18%), nausea (14%), and myalgia (11%). The most common adverse events of rituximab were infusion reaction (2%), upper respiratory tract infection (1%), herpes zoster infection (1%), and enteritis (1%). Preventive non-steroid anti-inflammatory drugs may be considered for eculizumab treatment, and management of mild adverse events were important for adherence.

The efficacy and safety of low-dose and high-dose rituximab were also compared. High-dose rituximab was defined as a dose higher than 375 mg/m^2^ two times a month. High-dose rituximab had a higher MM rate than low-dose rituximab (84 vs. 39%, Figure 6A), while no significant difference was observed between their adverse event rates (Figure 6B). In refractory MG, high-dose rituximab appears to be a better regimen. However, the number of included rituximab studies was limited, and their regimens differed. A head-to-head comparison is needed to support the advantage of high-dose rituximab.

The efficacy of tacrolimus and cladribine was also revealed in this study; however, the number of included studies was insufficient.

These studies have several limitations. First, most of these were observational studies without control. The quality of those observational studies varied, and few high-quality studies were included. Second, the number of included patients was not enough, especially that of tacrolimus and cladribine. Moreover, the reported outcome of each study differed from each other, resulting in the insufficiency of the included patients in the quantitative synthesis. More controlled studies with standardized outcomes are necessary to search for the optimal treatment of refractory MG.

There was a growing number of new options for treatment of refractory MG, including neonatal Fc receptor blocking agents (37), Bortezomib [a proteasome inhibitor (38)], tocilizumab [blocker of interleukin-6 (39)], etc. However, few of them had been tested in trials of refractory MG. More well-designed clinical trials of these treatments on refractory MG and vigorous systemic reviews should be considered in order to establish an effective standardized treatment for patients with refractory MG.

This study revealed the efficacy and safety of eculizumab and rituximab in patients with refractory MG. Although few of the adverse events of eculizumab were serious, they were common to some degree during eculizumab treatment. Certain preventions may be necessary for better long-time adherence.

## Data Availability Statement

The raw data supporting the conclusions of this article will be made available by the authors, without undue reservation.

## Author Contributions

Article search was performed by XF. Full-text screening was performed by XF and YL. Quality evaluation was performed by XF and ZS. Data extract was performed by XF, MW, and WZ. Manuscript was written by XF, SL, and CZ. Study was designed by WZ. All authors contributed to the article and approved the submitted version.

## Funding

This study was supported by grants from the National Key Research and Development Program (2018YFC1311304, 2018YFC1311300), the Southern China International Cooperation Base for the Early Intervention and Functional Rehabilitation of Neurological Diseases (2015B050501003), Guangdong Provincial Engineering Center for Major Neurological Disease Treatment, Guangdong Provincial Translational Medicine Innovation Platform for Diagnosis and Treatment of Major Neurological Disease, and Guangdong Provincial Clinical Research Center for Neurological Diseases. The sponsor or funding organization had no role in the design or conduct of this research.

## Conflict of Interest

The authors declare that the research was conducted in the absence of any commercial or financial relationships that could be construed as a potential conflict of interest.

## Publisher's Note

All claims expressed in this article are solely those of the authors and do not necessarily represent those of their affiliated organizations, or those of the publisher, the editors and the reviewers. Any product that may be evaluated in this article, or claim that may be made by its manufacturer, is not guaranteed or endorsed by the publisher.
