# Supplementary figures and images for: Efficacy and Safety of Immunotherapies in Refractory Myasthenia Gravis: A Systematic Review and Meta-Analysis
Source: Front Neurol. 2021 Dec 1;12:725700. doi: 10.3389/fneur.2021.725700 (PMC8672452; doi:10.3389/fneur.2021.725700)

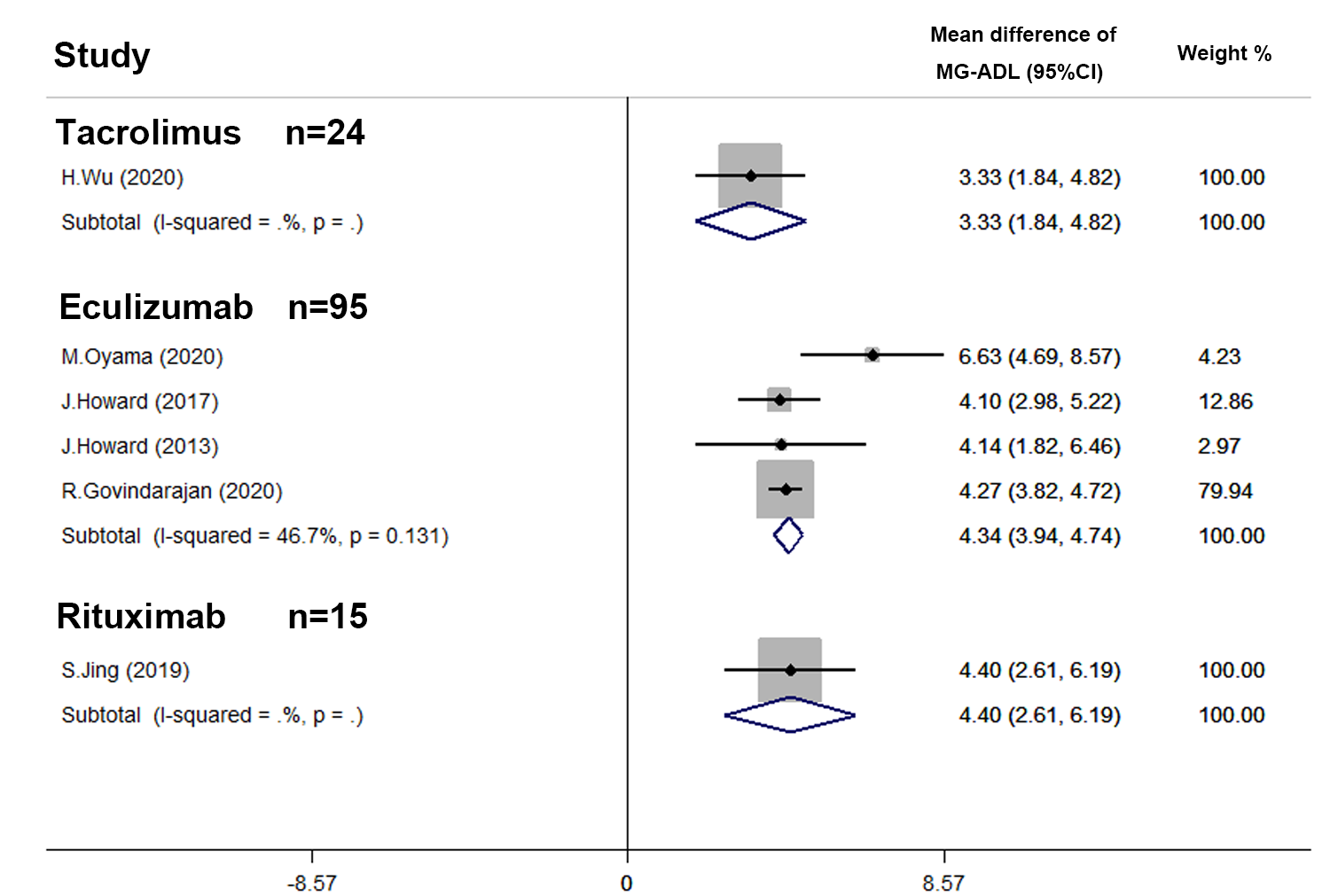

Supplement: Supplementary file 1 [file Image_1.TIF]

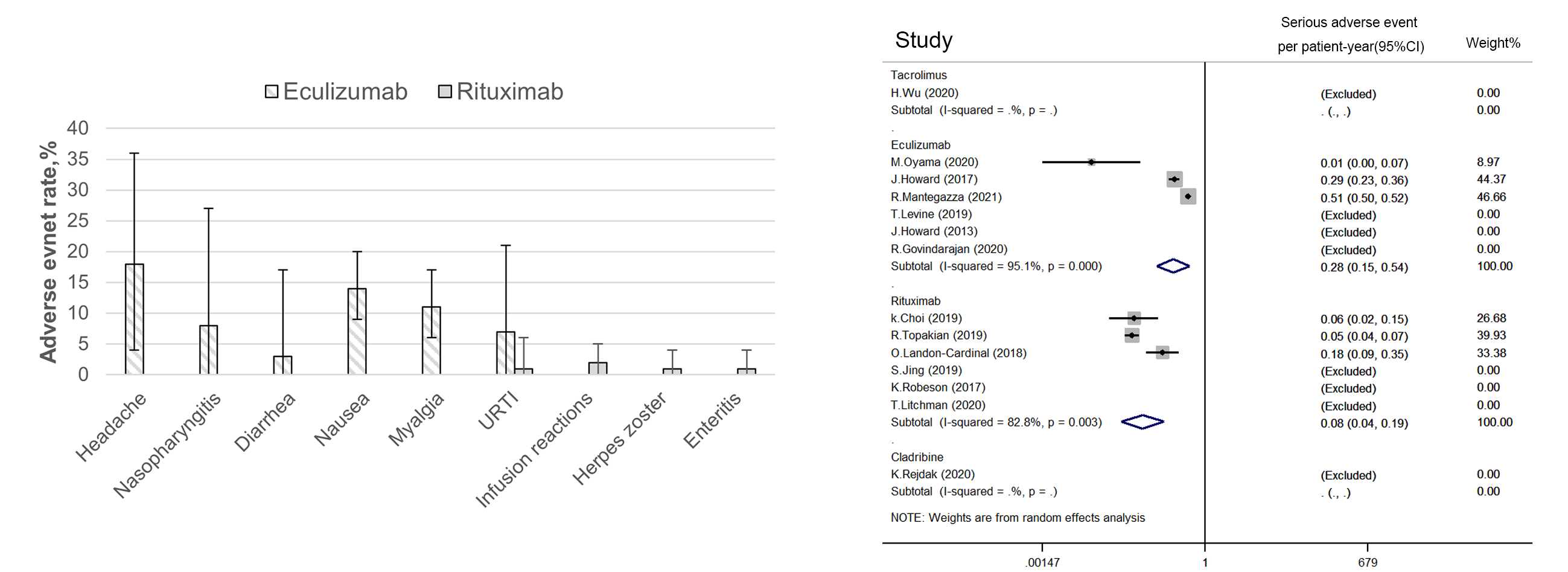

Supplement: Supplementary file 2 [file Image_2.TIF]
