# Supplementary material for: Efficacy and Safety of Immunotherapies in Refractory Myasthenia Gravis: A Systematic Review and Meta-Analysis
Source: Front Neurol. 2021 Dec 1;12:725700. doi: 10.3389/fneur.2021.725700 (PMC8672452; doi:10.3389/fneur.2021.725700)
Supplement: Supplementary file 5 [file Data_Sheet_1.docx]

Supplemental figure.1 Efficacy of immunotherapy for refractory myasthenia gravis based on Myasthenia Gravis Activities of Daily Living scale reduction.

Supplemental figure.2. A. Incidence of common adverse event of rituximab and eculizumab. B. Serious adverse event density(event rate per patient-year) of rituximab, eculizumab, tacrolimus and cladribine. Serious adverse events were adverse events that are life-threatening or result in death, hospitalization, or persistent or significant disability or incapacity, are congenital anomalies or birth defects, or are important medical events

Supplemental table S1.Quality assessment of selected studies.

Supplemental table S2. Summary of included studies assessed by full-text review.
